# Mapping Digital Public Health Interventions Among Existing Digital Technologies and Internet-Based Interventions to Maintain and Improve Population Health in Practice: Protocol for a Scoping Review

**DOI:** 10.2196/33404

**Published:** 2022-03-31

**Authors:** Laura Maaß, Chen-Chia Pan, Merle Freye

**Affiliations:** 1 Department of Health, Long-Term Care and Pensions Research Center on Inequality and Social Policy Bremen Germany; 2 Leibniz ScienceCampus Digital Public Health Bremen Bremen Germany; 3 Department for Epidemiological Methods and Etiological Research Leibniz Institute for Prevention Research and Epidemiology Bremen Germany; 4 Institute for Information, Health and Medical Law Bremen Germany

**Keywords:** digital public health, telemedicine, electronic health records, ePrescription, eReferral, eConsultation, eSurveillance, eVaccination registries, scoping review, protocol

## Abstract

**Background:**

Rapid developments and implementation of digital technologies in public health domains throughout the last decades have changed the landscape of health delivery and disease prevention globally. A growing number of countries are introducing interventions such as online consultations, electronic health records, or telemedicine to their health systems to improve their populations’ health and improve access to health care. Despite multiple definitions for digital public health and the development of different digital interventions, no study has analyzed whether the utilized technologies fit the definition or the core characteristics of digital public health interventions. A scoping review is therefore needed to explore the extent of the literature on this topic.

**Objective:**

The main aim of this scoping review is to outline real-world digital public health interventions on all levels of health care, prevention, and health. The second objective will be the mapping of reported intervention characteristics. These will include nontechnical elements and the technical features of an intervention.

**Methods:**

We searched for relevant literature in the following databases: PubMed, Web of Science, CENTRAL (Cochrane Central Register of Controlled Trials), IEEE (Institute of Electrical and Electronics Engineers) Xplore, and the Association for Computing Machinery (ACM) Full-Text Collection. All original study types (observational studies, experimental trials, qualitative studies, and health-economic analyses), as well as governmental reports, books, book chapters, or peer-reviewed full-text conference papers were included when the evaluation and description of a digital health intervention was the primary intervention component. Two authors screened the articles independently in three stages (title, abstract, and full text). Two independent authors will also perform the data charting. We will report our results following the PRISMA-ScR (Preferred Reporting Items for Systematic Reviews and Meta-Analyses Extension for Scoping Reviews) checklist.

**Results:**

An additional systematic search in IEEE Xplore and ACM, performed on December 1, 2021, identified another 491 titles. We identified a total of 13,869 papers after deduplication. As of March 2022, the abstract screening state is complete, and we are in the state of screening the 1417 selected full texts for final inclusion. We estimate completing the review in April 2022.

**Conclusions:**

To our knowledge, this will be the first scoping review to fill the theoretical definitions of digital public health with concrete interventions and their characteristics. Our scoping review will display the landscape of worldwide existing digital public health interventions that use information and communication technologies. The results of this review will be published in a peer-reviewed journal in early 2022, which can serve as a blueprint for the development of future digital public health interventions.

**International Registered Report Identifier (IRRID):**

DERR1-10.2196/33404

## Introduction

### Background

The potential of digital technology for improving the health of individuals, communities, and populations is unprecedented. Technological advancements empower individuals to engage in self-management and well-being [1]. There is also the unparalleled opportunity of digital technologies to transform the prevention [2], health promotion [3,4], health monitoring [5], health management [6,7], health equity [8], and surveillance for public health disasters [9-11].

Digital technologies for health are intrinsically interdisciplinary, including computer science, engineering, information science, clinical medicine, epidemiology, and public health [12,13]. Although these disciplines are involved at various stages, from the development process to the implementation of digital technologies, a shared understanding of key terms within the field of digitalization in health is still missing. More importantly, to develop, implement, integrate, and evaluate needs-based digital public health interventions (DiPHIs), a clear and mutual understanding of the specific properties of digital health technologies for public health purposes is required [14].

### Definition of Digital Public Health

For this review, we will define digital public health following the understanding of the European Public Health Association as the use of digital technologies or tools to achieve public health goals. Therefore, digital public health is not a new discipline but rather represents the digitalization of public health [15]. Following the definition of Winslow [16], public health aims at “preventing disease, prolonging life, and promoting physical [and mental] health and efficiency through organized community efforts.” These include health education, organizing health care (both medical and nursing services), or controlling infections on a community level (or on a global level as evident during the COVID-19 pandemic) from an individual to a community, national, or international level [16,17]. Although the definition provided by Winslow is over 100 years old, it is still the most referred to definition of public health. For instance, the World Health Organization still uses this definition with respect to essential public health functions. These functions place public health as the primary discipline in health governance (eg, planning of health services), financing health interventions and services, health information systems (eg, surveillance systems), health communication, universal health coverage, health education, or health regulations to protect and ensure the health of vulnerable groups [18].

This importance of the given definition of public health makes it suitable for our understanding of digital public health as the digitalization of Winslow’s definition. According to the ongoing importance of Winslow’s description in essential public health functions, we define DiPHIs as interventions that address “at least one essential public health function through digital means” [19]. Similar to public health interventions, DiPHIs aim to strengthen the population’s health from the individual to the community and national levels. To achieve this, DiPHIs, similar to interventions for digital health, eHealth, or mobile health (mHealth), use information and communication technologies (ICTs) [12,20]. ICTs include the use of radio, television, smartphones, hardware, and software for computers and satellites for communication purposes. eHealth is defined as the use of ICT for health purposes, whereas the focus lies on delivering health services and not on health promotion itself [21,22]. mHealth is understood as using wireless and mobile technology to improve health [21]. The main difference between digital public health and digital health (including eHealth and mHealth) is that the former solely targets individuals’ health, whereas digital public health targets groups of people and communities [14]. With the combination of public health goals (defined as the above-mentioned essential public health functions [18]) and ICT application, DiPHIs can monitor public health outcomes and disasters (as seen in tracing apps for the COVID-19 pandemic) [23]. In the best case, DiPHIs follow an evidence- and needs-based approach with a participatory user–targeted development design to improve the acceptance of the intervention within the population [12,14,19,24,25].

**Figure 1:**
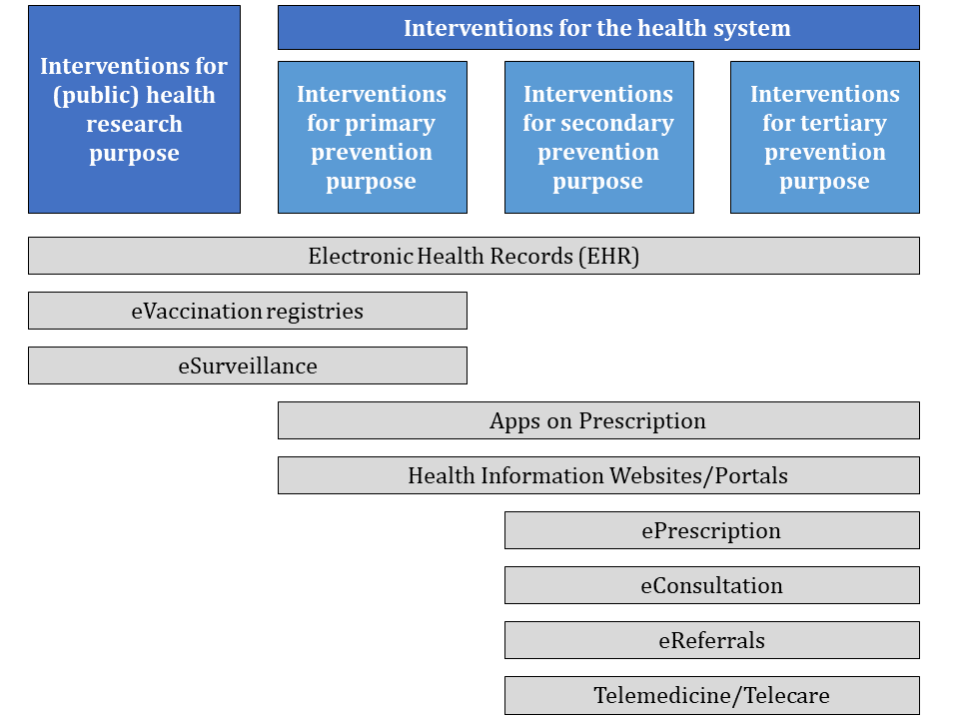
The proposed landscape of digital public health intervention classification.

We expect that the described interventions of our finally chosen papers will target one of the health areas listed in Figure 1. Nevertheless, we will likely identify other DiPHIs throughout the review process and update this first proposed landscape of interventions in digital public health.

### Study Aim and Objective

This scoping review aims to serve as a blueprint for future DiPHIs to support countries in adapting digital public health to their health system. To fulfill this aim, the main objective is to outline real-world DiPHIs on all levels of health care (primary, secondary, and tertiary health care), prevention, and health promotion based on our predefined definition of a DiPHI. The second objective will be to map reported intervention characteristics of existing real-world DiPHIs.

For this scoping review, intervention characteristics will include the nontechnical elements (eg, the target group or the addressed level of health care) and the technical features of an intervention (eg, data exchange between health providers). Our review will not provide a detailed analysis of the cost-effectiveness of DiPHIs, their influence on health outcomes, the facilitators or barriers for implementation, or adaptation of interventions, as DiPHIs overall are too heterogeneous to be summarized within one literature review [26,27].

## Methods

### Inclusion Criteria

#### Overview

The inclusion criteria follow the PIOS (Participants, Intervention, Outcome, and Study design) format. The inclusion and exclusion criteria are summarized in Table 1 and described in detail below.

**Table 1:** Inclusion and exclusion criteria of the scoping review.

Level	Inclusion criteria	Exclusion criteria
Population	The study focuses on the geographical community level or above (regional or national)	The study population consists of veterans, armed forces, prisoners, inmates, refugees, or asylum seekers
Intervention	The paper describes a concrete DiPHI^a^; the DiPHI is paid or reimbursed by the government or health insurance; the intervention uses the internet and/or Bluetooth to facilitate health care, allows communication between providers or providers and patients, promotes its users’ health, reuses the collected data for public health research, or enables digital surveillance of public health disasters	The paper offers a framework or overview of an intervention type but does not describe a concrete intervention in detail; the intervention does not use the internet, does not address public health functions, or needs to be privately bought by the user without reimbursement by the government or health insurance (ie, interventions for the private market); the intervention uses SMS text messaging or regular phone calls; the intervention focuses on background management processes; the described intervention does not match our definition of digital public health; the intervention targets only the individual user but not a group of people; the digital public health intervention is not the central research object of the publication
Study design	All original peer-reviewed studies, reports, books, book chapters, or peer-reviewed conference papers that have a description of a DiPHI as their primary intervention component	Study protocols, editorials, commentaries, conference proceedings, or reviews (narrative reviews, scoping reviews, systematic reviews, or meta-analyses); not peer-reviewed conference papers or original studies
Accessibility	The paper is available on the internet or after contact with the authors	The full text is not available on the internet or after contact with the authors
Language	The paper is published in English, Chinese, or German	The paper is published in a language other than English, Chinese, or German

^a^DiPHI: digital public health intervention.

#### Study Populations/Participants

We will extract data from all studies that focus on the geographical community level and above (eg, regional or national). As public health concentrates on the population level and not the merely individual level, we will exclude all case studies that focus exclusively on single institutions or departments (eg, single hospitals or emergency departments) but do not address at least the community level.

Our scoping review will aim to describe DiPHIs and online technologies for the general public (ordinary citizens of a city/state country that form a society). We will include papers addressing health care workers (eg, physicians, nurses, or therapists) and people with access to health care and health insurance (both statutory and private health insurance) without further regulations or restrictions. In contrast, we will exclude groups with limited or special access to public health care (ie, having their own health care system). This applies to the following three groups with precisely regulated access to health care: (1) veterans and the armed forces (who receive treatment in many countries within the military health system, often paid by the ministry of defense), (2) prisons and inmates (for which a prison physician needs to approve the treatment by another physician outside the prison), and (3) refugees and asylum seekers (who are not health insured in most countries during the procedure for granting the right of asylum and therefore often only have limited access to health care).

Our review will include all participants in terms of age, gender, ethnicity, morbidities, education level, staff role, and occupation. We will include studies that examined specific interventions among health care providers (eg, physicians, nurses, or therapists) and studies that analyzed the use of interventions by laypersons (people with a profession outside medicine or health care or with no profession).

#### Interventions/Technologies

Following our definition of a DiPHI, we will include studies describing digital health interventions that use the internet (eg, mobile devices such as smartphones, sensors, or wearables with WiFi; computer-based solutions that use cellular services; cloud systems to store/allocate health data; or wireless medical devices) and/or Bluetooth (eg, mobile devices such as smartphones, sensors, or wearables) to address at least one essential public health function to (1) facilitate health care, (2) allow for communication between providers or between providers and patients, (3) promote one’s health, (4) collect data in a way that enables their secondary use in public health research, or (5) allow for digital surveillance of public health disasters.

We will exclude every digital health intervention that does not address one of the five inclusion criteria listed above as they are not deemed to represent digital public health based on our definition.

To be included, studies should not exclusively focus on individuals (as is the case for digital health interventions) but should also focus on groups of people (eg, communities). They will also have to report on the DiPHI as the main object of research and offer a description of the intervention/technology. Such examples include studies explaining the implementation of a national digital surveillance system in a country or the social acceptance of online consultations in a public health system.

Studies that use a DiPHI only for secondary data analysis but do not have the DiPHI as the main object of research will also be omitted. Examples of such papers are studies mainly interested in measuring obesity in a community and only use electronic health records (EHRs) as a data source without going into detail on the records themselves. Therefore, the EHR in such study types would not be seen as an intervention for the sake of this scoping review. We will also exclude studies that offer no precise details on the modules of the intervention or its implementation process, such as studies that only give a brief overview of telemedicine opportunities in general without a concrete adaptation case. We will further exclude studies that evaluate or present interventions for the private market, for which individuals will have to buy without the option of a refund from their public health system (eg, privately bought wearables or apps that are not provided/refunded by a public health institution, insurance, or the government). Interventions that do not use the internet or Bluetooth (eg, a regular phone call for remote counseling or SMS as text reminder interventions) will be excluded. Technologies that focus solely on background management processes (eg, hospital management systems) are not considered to be DiPHIs and are therefore not included.

#### Outcome Measures

To answer our research questions, we will map the description of DiPHIs and their characteristics presented in the selected papers. These elements will be clustered to form an overview of specific modules for DiPHIs.

#### Study Designs

The scoping review will include all suitable published papers to gather information on all digital tools classified as DiPHIs. This includes all peer-reviewed original studies (observational studies, experimental trials, qualitative studies, and health-economic analyses), governmental reports, books, book chapters, or peer-reviewed conference papers that have the evaluation and description of a digital health intervention as their primary intervention component.

We will exclude study protocols, editorials, and commentaries based on their limited containment of an intervention description and concrete original study results. Further, we will exclude review study types such as narrative, scoping, and systematic reviews and meta-analyses to avoid duplications. We will also exclude conference proceedings and not peer-reviewed publications (original studies and conference papers). Lastly, publications will be excluded in the screening process if the described intervention does not match our definition of digital public health.

### Literature Search

**Figure 2:**
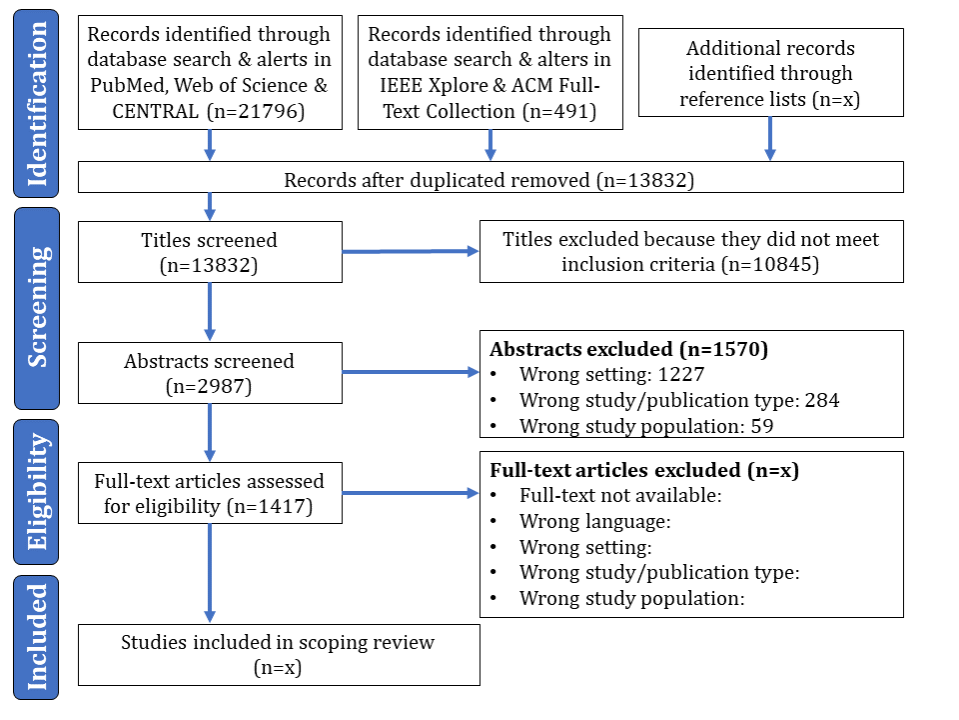
Flow chart of the search and screening process. ACM: Association for Computing Machinery; CENTRAL: Cochrane Central Register of Controlled Trials; IEEE: Institute of Electrical and Electronics Engineers.

We will use a two-part search strategy to identify publications that meet our inclusion criteria. For the first part, we searched three electronic bibliographic databases for published work on February 19, 2021, using a comprehensive search strategy for possible DiPHIs: PubMed, CENTRAL (Cochrane Central Register of Controlled Trials), and Web of Science (see Figure 2, left box in the identification phase). We added two databases, IEEE (Institute of Electrical and Electronics Engineers) Xplore and the Association for Computing Machinery (ACM) Full-Text Collection, to the systematic search to identify more publications from computer science. Both databases were searched on December 1, 2021 (see Figure 2, middle box in the identification phase). All five search strategies are based on the PubMed search string but have been adapted to consider differences in the vocabulary used by the database (Medical Subject Heading [MeSH] terms) and its syntax specifications. To ensure that the systematic search results are not outdated by the time of data extraction, we set up alerts for all databases about new publications on the topic of interest. We will include all alerts that fit our inclusion criteria and that appeared until the start of the full-text screening. For the second part of our search strategy, we will manually screen the reference lists of studies included in the scoping review (see Figure 2, right box in the identification phase). This will ensure that relevant studies are not overlooked.

**Figure 3:**
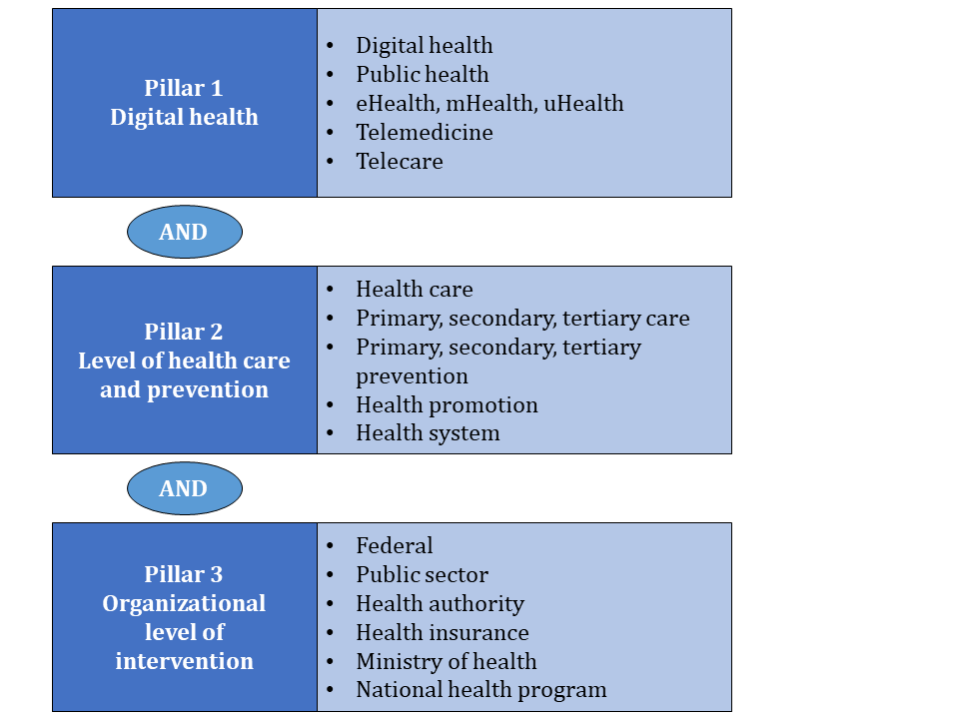
Short version of the search strategy. mHealth: mobile health.

The search strategy for each database consists of three pillars, each combined with “AND.” Individual terms within the bodies are connected with “OR.” The first pillar includes terms related to digital health and the second contains different health care and prevention levels. The third pillar finally lists search terms to describe the organizational level of interventions (see Figure 3 for the short version and see Multimedia Appendix 1 for the extended version for all five databases). We decided against naming concrete interventions such as EHR, mHealth apps, or electronic prescriptions to reduce the risk of confirmation bias.

For PubMed and CENTRAL, the search bodies also included MeSH terms to identify publications listed within those categories. All search terms will be limited to title, abstract, and keywords. There will be no use of additional filters such as language, geographical, or year of publication restrictions. Exclusion of publications due to another language will be made during full-text screening. Our scoping review will not have any unpublished paper or grey literature.

Identified studies will be screened independently by two authors (LM and MF). Following the aforementioned inclusion criteria, we will separate the screening process into title screening, abstract screening, and full-text screening. In case of disagreements over the eligibility of specific papers, the two screening authors (LM and MF) will discuss whether or not to include a publication for each of the three stages of the screening process (title, abstract, and full-text screening). If the disagreement cannot be resolved through discussion, the third author (CCP) will have the final decision on the eligibility of the publication. We will calculate the Cohen κ value for each step of a screening process to illustrate the agreement between the two screening authors (LM and MF).

### Data Extraction

Following quality assessment, we will extract data from the included studies. Two review authors (LM and CCP) will independently extract data in a Microsoft Excel 2019 sheet. These data will contain information on: (1) participants, including recruitment, study completion rates, study population, and participant demographics (age, gender, insurance status, race, ethnicity, education level, income, geography, and language); (2) intervention, including a description of the intervention, target group, use case, addressed level of prevention/health care, geographical level of intervention (local, regional, national), and core function/modules of the intervention; (3) outcomes, including indicators of user acceptability, indicators of health economic evaluation, and other outcomes and indicators; and (4) setting, including the country, year of publication, publication type, study methodology, and year of data collection.

We will request missing data from the authors of our included studies via email. We will resolve any discrepancies through discussion with the third author, MF.

### Synthesis of Results

All included studies will be summarized in a narrative synthesis. We will group all included papers in a table according to the described intervention type (see Figure 1). Based on the interventions’ descriptions, we will map out each intervention type’s characteristics and technological functions. As an example, many countries offer an EHR system at the national level. However, most of them have a different understanding of EHRs (which makes empirical research on this issue complicated). Our scoping review will therefore extract characteristics of the EHR from one publication (reporting on a whole country or a state/region within a county) and compare those with the attributes of an EHR from a different report (country, state, region). This approach will result in overlaps of characteristics, which can be defined as core characteristics if the majority of all EHRs share this attribute. Some EHRs might have features that none or only a few others have. These can be defined as “added characteristics.” To display common functions within each intervention type, we will create a table with all mentioned characteristics and rank each intervention based on how many features it includes (see Table 2).

**Table 2:** Example table of core characteristics for a given intervention group based on electronic health records.

Study	Characteristic 1	Characteristic 2	Characteristic 3	Characteristic 4
Study 1	✓	✓	✓	
Study 2		✓		✓
Study 3	✓		✓	✓

For each intervention type, we will analyze the following: the country setting by income level according to the World Bank [28], level of prevention and health care (primary, secondary, or tertiary prevention), target group (eg, medical provider, health insurance, researchers, or general population), and use case (eg, communication facilitator, health education, tracking, tracing, surveillance, or self-management of chronic disease).

We will further analyze differences between locally implemented interventions and national digital public health policies for the same intervention group. The clusters for categorization of the interventions’ features will be developed during data extraction based on our research evidence. This approach is used mainly in qualitative research. Based on the aim of our scoping review, we decided against predefined clusters for categorization as these would require predefined descriptions of intervention characteristics, which we want to explore with this review.

## Results

The systematic search in the three databases, Web of Science, CENTRAL, and PubMed, was performed on February 19, 2021, and produced 18,363 results. A total of 13,383 papers were included in the review after deduplication. Of these, 2962 titles were included for abstract screening. We performed an additional systematic search in the IEEE Xplore and ACM Full-Text Collection databases on December 1, 2021, which identified another 491 titles, 38 of which were included for abstract screening. In addition, 22 abstracts from the second search were included for full-text screening. In total, we have included 1417 publications for full-text screening and expect to complete this scoping review in April 2022. The results will be published in peer-reviewed journals and conferences based on the identified outcomes.

## Discussion

### General Aims and Significance

This paper presents the protocol for a scoping review following the PRISMA-ScR (Preferred Reporting Items for Systematic Reviews and Meta-Analyses Extension for Scoping Reviews) reporting standards [29]. This scoping review of DiPHIs will provide an extensive report of the literature about worldwide existing DiPHIs that take advantage of ICT. To our knowledge, no review has been conducted with the broad scope proposed here. We will show what characteristics they have in common and the areas they cover from a population-health level. This information will be useful as a blueprint for future DiPHI development to support countries in adapting digital public health to all areas of their health system.

### Strengths

The chosen method of a scoping review is an effective technique for mapping comprehensive and interdisciplinary topics such as digital public health. The search is performed in the three largest databases for digital public health topics (ie, PubMed, CENTRAL, and the Web of Science) and the two largest databases for computer science (IEEE Xplore and ACM Full-Text Collection), which reduces the risk of missed articles. Two researchers (LM and MF) will systematically and independently select the studies that fit the inclusion criteria. The third author (CCP) will settle disagreements to ensure the reliability of the results. Additionally, we explained our study identification process in detail, including inclusion criteria, to ensure reproducibility. The review will follow the PRISMA-ScR checklist that is specific for scoping reviews [29] to ensure the high level of quality and transparency of this study.

### Limitations

One limitation of our scoping review is that we do not include study protocols, editorials, or commentaries. It is also worth mentioning that although we include research performed worldwide, our review is limited to publications in English, German, or Chinese. This could limit the completeness of the identified publications. Publications might also be missed as we will restrict the manual search to the reference lists of included studies. We will not assess the quality of the included literature in this review, which may lead to concerns about the accuracy of the literature and affect the generalizability and evidence of the results. However, as we are not assessing the outcome results of the selected studies but only examining their description of a DiPHI, the missing quality analysis is not relevant to our scoping review. The last limitation is that this review is not going to assess the impact of the implementation of DiPHIs in a health system, but will rather only provide an overview of existing technologies and their characteristics.

## Appendix

Multimedia Appendix 1 Complete search strategies for the PubMed, Web of Science, CENTRAL, IEEE Xplore, and ACM Full-Text Collection databases.

## Abbreviations

ACM: Association for Computing Machinery
CENTRAL: Cochrane Central Register of Controlled Trials
DiPHI: digital public health intervention
EHR: electronic health record
ICT: information and communication technology
IEEE: Institute of Electrical and Electronics Engineers
MeSH: Medical Subject Headings
mHealth: mobile health
PIOS: Participants, Intervention, Outcome, and Study design
PRISMA-ScR: Preferred Reporting Items for Systematic Reviews and Meta-Analyses extension for Scoping Reviews
