# Supplementary material for: Mapping Digital Public Health Interventions Among Existing Digital Technologies and Internet-Based Interventions to Maintain and Improve Population Health in Practice: Protocol for a Scoping Review
Source: JMIR Res Protoc. 2022 Mar 31;11(3):e33404. doi: 10.2196/33404 (PMC9015775; doi:10.2196/33404)
Supplement: Multimedia Appendix 1 [file resprot_v11i3e33404_app1.docx]

**Additional File 1: Complete Search Strategies PubMed, Web of Science, CENTRAL, Ieee Xplore, and ACM Full-Text Collection**

| **No** | **Medline (PubMed)**  **(19th Feb 12:40 PM CET)** | | **Web of Science**  **(19th Feb 12:40 PM CET)** | | **CENTRAL**  **(19th Feb 12:40 PM CET)** | | **Ieee Xplore**  **(1st Dec 2:00 PM CET)** | | **ACM Full-Text Collection**  **(1st Dec 2:00 PM CET)** | |
| --- | --- | --- | --- | --- | --- | --- | --- | --- | --- | --- |
|  | **Search Term** | **Results** | **Search Term** | **Results** | **Search Term** | **Results** | **Search Term** | **Results** | **Search Term** | **Results** |
| #1 | “digital health”  [Title/Abstract] | 2.683 | TS=("digital health") | 2.999 | (“digital health”):ti,ab | 260 | "Document Title":"digital health" OR "Abstract":"digital health" | 207 | Title:("digital health") OR Abstract:("digital health") | 251 |
| #2 | “digital public health”  [Title/Abstract] | 20 | TS=("digital public health") | 29 | (“digital public health”):ti,ab | 0 | "Document Title":"digital public health" OR "Abstract":"digital public health" | 1 | Title:("digital public health") OR Abstract:("digital public health") | 43 |
| #3 | “health 2.0“  [Title/Abstract] | 69 | TS=("health 2.0") | 129 | (“health 2.0”):ti,ab | 3 | "Document Title":"health 2.0" OR "Abstract":"health 2.0" | 13 | Title:("health 2.0") OR Abstract:("health 2.0") | 10 |
| #4 | Mhealth  [Title/Abstract] | 5.895 | TS=(mhealth) | 6.732 | (mhealth):ti,ab | 1.266 | "Document Title":mhealth OR "Abstract": mhealth | 571 | Title:(mhealth) OR Abstract:(mhealth) | 359 |
| #5 | m-health  [Title/Abstract] | 641 | TS=(m-health) | 1.285 | (m-health):ti,ab | 1.255 | "Document Title":"m health" OR "Abstract":"m health" | 330 | Title:("m health") OR Abstract:("m health") | 35 |
| #6 | “m health”  [Title/Abstract] | 641 | TS=("m health") | 1.285 | (“m health”):ti,ab | 1.718 | "Document Title":"mobile health" OR "Abstract":"mobile health" | 815 | Title:("mobile health") OR Abstract:("mobile health") | 250 |
| #7 | “mobile health”[Title/Abstract] | 5.197 | TS=("mobile health") | 6.655 | (“mobile health”):ti,ab | 1.177 | "Document Title":ehealth OR "Abstract": ehealth | 760 | Title:(ehealth) OR Abstract:(ehealth) | 122 |
| #8 | mobile-health  [Title/Abstract] | 5.197 | TS=(ehealth) | 6.583 | (mobile-health):ti,ab | 1.177 | "Document Title":"e health" OR "Abstract":"e health" | 1.830 | Title:("e health") OR Abstract:("e health") | 218 |
| #9 | Ehealth[Title/Abstract] | 6.560 | TS=(e-health) | 7.517 | (ehealth):ti,ab | 955 | "Document Title":"electronic health" OR "Abstract":"electronic health" | 1.867 | Title:("electronic health") OR Abstract:("electronic health") | 463 |
| #10 | e-health  [Title/Abstract] | 3.306 | TS=("e health") | 7.517 | (e-health):ti,ab | 967 | "Document Title":uhealth OR "Abstract": uhealth | 3 | Title:(uhealth) OR Abstract:(uhealth) | 0 |
| #11 | “e health”  [Title/Abstract] | 3.306 | TS=("electronic health") | 22.517 | ("e health"):ti,ab | 1.105 | "Document Title":"u health" OR "Abstract":"u health" | 67 | Title:("u health") OR Abstract:("u health") | 6 |
| #12 | “electronic health”  [Title/Abstract] | 20.372 | TS=(uhealth) | 23 | (“electronic health”):ti,ab | 1.911 | "Document Title":"ubiquitous health" OR "Abstract":"ubiquitous health" | 113 | Title:("ubiquitous health") OR Abstract:("ubiquitous health") | 20 |
| #13 | uhealth[Title/Abstract] | 34 | TS=(u-health) | 170 | (uhealth):ti,ab | 7 | "Document Title":Telemedicine OR "Abstract": Telemedicine | 2.728 | Title:(Telemedic*) OR Abstract:(Telemedic*) | 152 |
| #14 | u-health  [Title/Abstract] | 32 | TS=("ubiquitous health") | 253 | (u-health):ti,ab | 9 | "Document Title":Telecare OR "Abstract": Telecare | 251 | Title:(Telecar*) OR Abstract:(Telecar*) | 38 |
| #15 | “u health“  [Title/Abstract] | 32 | TS=(telemedicine) | 23.838 | ("u health"):ti,ab | 9 | "Document Title":telehealth OR "Abstract": telehealth | 603 | Title:(telehealth) OR Abstract:(telehealth) | 52 |
| #16 | “ubiquitous health“  [Title/Abstract] | 80 | TS=(telecare) | 1.745 | (“ubiquitous health”):ti,ab | 2 | "Document Title":telemonitoring OR "Abstract": telemonitoring | 432 | Title:(telemonitor*) OR Abstract:(telemonitor*) | 21 |
| #17 | Telemedicine  [Title/Abstract] | 15.682 | TS=(telehealth) | 8.869 | (Telemedicine):ti,ab | 3.858 | "Document Title":"Remote Consultation" OR "Abstract":"Remote Consultation" | 34 | Title:("Remote Consultation") OR Abstract:("Remote Consultation") | 5 |
| #18 | Telecare  [Title/Abstract] | 817 | TS=(telemonitoring) | 2.570 | (Telecare):ti,ab | 182 | "Document Title":"Distance Counseling" OR "Abstract":"Distance Counseling" | 1 | Title:("Distance Counseling") OR Abstract:("Distance Counseling") | 0 |
| #19 | Telehealth  [Title/Abstract] | 6.827 | TS=(telecommunication*) | 58.182 | (telehealth):ti,ab | 1.865 | **{OR #1-18}** | **9.245** | **{OR #1-#18}** | **1.659** |
| #20 | Telemonitoring  [Title/Abstract] | 1.812 | TS=("remote consultation") | 510 | (telemonitoring):ti,ab | 1.016 | "Document Title":"health care" OR "Abstract":"health care" | 8.578 | Title:("health care") OR Abstract:("health care") | 1.602 |
| #21 | telecommunication* [Title/Abstract] | 4.580 | TS=("distance counseling") | 29 | (telecommunication*):ti,ab | 534 | "Document Title":healthcare OR "Abstract":healthcare | 18.968 | Title:("healthcare") OR Abstract:("healthcare") | 4.409 |
| #22 | “telemedicine“[Mesh] | 32.661 | **{OR #1-#21}** | **131.880** | MeSH descriptor: [Telemedicine] explode all trees | 2.649 | "Document Title":"health system" OR "Abstract":"health system" | 711 | Title:("health system") OR Abstract:("health system") | 130 |
| #23 | “Remote Consultation“[Mesh] | 5.102 | TS=("health care") | 406.041 | MeSH descriptor: [Remote Consultation] explode all trees | 391 | "Document Title":"health systems" OR "Abstract":"health systems" | 633 | Title:("health systems") OR Abstract:("health systems") | 166 |
| #24 | “Distance Counseling“[Mesh] | 66 | TS=(healthcare) | 257.720 | MeSH descriptor: [Distance Counseling] explode all trees | 20 | "Document Title":"primary care" OR "Abstract":"primary care" | 335 | Title:("primary care") OR Abstract:("primary care") | 70 |
| #25 | **{OR #1-#24}** | **74.185** | TS=(health-care) | 406.162 | **{OR #1-#24}** | **11.317** | "Document Title":"secondary care" OR "Abstract":"secondary care" | 13 | Title:("secondary care") OR Abstract:("secondary care") | 6 |
| #26 | “health care“  [Title/Abstract] | 391.538 | TS=("health system") | 41.073 | (“health care”):ti,ab | 58.949 | "Document Title":"tertiary care" OR "Abstract":"tertiary care" | 53 | Title:("tertiary care") OR Abstract:("tertiary care") | 6 |
| #27 | “healthcare“  [Title/Abstract] | 263.891 | TS=("primary care") | 147.519 | (“healthcare”):ti,ab | 21.011 | "Document Title":prevent OR "Abstract":prevent | 62.222 | Title:(prevent*) OR Abstract:(prevent*) | 11.634 |
| #28 | "health-care"  [Title/Abstract] | 391.538 | TS=("secondary care") | 7.055 | (“health-care”):ti,ab | 58.949 | "Document Title":"Health Promotion" OR "Abstract":"Health Promotion" | 173 | Title:("health promotion") OR Abstract:("health promotion") | 68 |
| #29 | “health system“  [Title/Abstract] | 41.299 | TS=("tertiary care") | 54.229 | (“health system”):ti,ab | 2.65 | "Document Title":"Health Information System" OR "Abstract":"Health Information System" | 178 | Title:("Health Information System") OR Abstract:("Health Information System") | 31 |
| #30 | “health systems“  [Title/Abstract] | 21.008 | TS=(prevent*) | 1.879.428 | (“health systems”):ti,ab | 922 | "Document Title":"Health Information Systems" OR "Abstract":"Health Information Systems" | 226 | Title:("Health Information Systems") OR Abstract:("Health Information Systems") | 73 |
| #31 | “primary care“  [Title/Abstract] | 122.013 | TS=("health promotion") | 42.355 | (“primary care”):ti,ab | 18.563 | **{OR #20-30}** | **88.754** | **{OR #20-#30}** | **17.309** |
| #32 | “secondary care“  [Title/Abstract] | 7.031 | TS=("health information system*") | 4.692 | (“secondary care”):ti,ab | 1.3 | "Document Title": government OR "Abstract":government | 27.706 | Title:(government*) OR Abstract:(government*) | 7.893 |
| #33 | “tertiary care“  [Title/Abstract] | 51.085 | **{OR #23-#33}** | **2.601.278** | (“tertiary care”):ti,ab | 6.329 | "Document Title":federal OR "Abstract":federal | 6.055 | Title:(federal*) OR Abstract:(federal*) | 1.305 |
| #34 | prevent*  [Title/Abstract] | 1.503.970 | TS=(government*) | 500.697 | (prevent*):ti,ab | 231.425 | "Document Title": national OR "Abstract": national | 45.812 | Title:(national*) OR Abstract:(national*) | 10.207 |
| #35 | “health promotion“  [Title/Abstract] | 34.692 | TS=(federal*) | 161.659 | (“health promotion”):ti,ab | 9.876 | "Document Title":"ministry of health" OR "Abstract":"ministry of health" | 221 | Title:("ministry of health") OR Abstract:("ministry of health") | 26 |
| #36 | “Delivery of Health Care“[Mesh] | 1.110.486 | TS=(national*) | 1.209.471 | MeSH descriptor: [Delivery of Health Care] explode all trees | 46.211 | "Document Title":"health ministry" OR "Abstract":"health ministry" | 26 | Title:("health ministry") OR Abstract:("health ministry") | 5.474 |
| #37 | “Health Information Systems“[Mesh] | 1.370 | TS=(ministr*) | 55.649 | MeSH descriptor: [Health Information Systems] explode all trees | 12 | "Document Title":"institute of health" OR "Abstract":"institute of health" | 48 | Title:("institute of health") OR Abstract:("institute of health") | 6 |
| #38 | “Primary Health Care“[Mesh] | 165.523 | TS=(institute*) | 550.479 | MeSH descriptor: [Primary Health Care] explode all trees | 7.352 | "Document Title":"health institute" OR "Abstract":"health institute" | 11 | Title:("health institute") OR Abstract:("health institute") | 3 |
| #39 | “Secondary Care“[Mesh] | 689 | TS=(department*) | 405.918 | MeSH descriptor: [Secondary Care] explode all trees | 39 | "Document Title":"health institutes" OR "Abstract":"health institutes" | 11 | Title:("health institutes") OR Abstract:("health institutes") | 0 |
| #40 | “Tertiary Healthcare“[Mesh] | 1.184 | TS=("health agenc*") | 5.229 | MeSH descriptor: [Tertiary Healthcare] explode all trees | 20 | "Document Title":"department of health" OR "Abstract":"department of health" | 140 | Title:("department of health") OR Abstract:("department of health") | 29 |
| #41 | “Primary Prevention“[Mesh] | 155.766 | TS=("health autorit*") | 6 | MeSH descriptor: [Primary Prevention] explode all trees | 4.185 | "Document Title":"health department" OR "Abstract":"health department" | 46 | Title:("health department") OR Abstract:("health department") | 14 |
| #42 | “Secondary Prevention“[Mesh] | 21.006 | TS=("public sector*") | 35.583 | MeSH descriptor: [Secondary Prevention] explode all trees | 3.179 | "Document Title":"health departments" OR "Abstract":"health departments" | 32 | Title:("health departments") OR Abstract:("health departments") | 8 |
| #43 | “Tertiary Prevention“[Mesh] | 167 | TS=("health insurance*") | 45.429 | MeSH descriptor: [Tertiary Prevention] explode all trees | 4 | "Document Title":"health agency" OR "Abstract":"health agency" | 13 | Title:("health agency") OR Abstract:("health agency") | 4 |
| #44 | “Health Promotion“[Mesh] | 78.808 | TS=("ministr* of health") | 15.080 | MeSH descriptor: [Health Promotion] explode all trees | 6.617 | "Document Title":"health agencies" OR "Abstract":"health agencies" | 38 | Title:("health agencies") OR Abstract:("health agencies") | 22 |
| #45 | **{OR #26-#44}** | **3.181.642** | TS=("health ministr*") | 1.107 | **{OR #26-#44}** | **329.463** | "Document Title":"health authority" OR "Abstract":"health authority" | 31 | Title:("health authority") OR Abstract:("health authority") | 6 |
| #46 | government*  [Title/Abstract] | 114.494 | TS=("institute* of health") | 20.482 | (government*):ti,ab | 4.113 | "Document Title":"health authorities" OR "Abstract":"health authorities" | 113 | Title:("health authorities") OR Abstract:("health authorities") | 23 |
| #47 | federal*[Title/Abstract] | 54.103 | TS=("health institute*") | 1.090 | (federal*):ti,ab | 2.100 | "Document Title":"health insurances" OR "Abstract":"health insurances" | 8 | Title:("health insurances") OR Abstract:("health insurances") | 0 |
| #48 | national*  [Title/Abstract] | 563.338 | TS=("department* of health") | 9.083 | (national*):ti,ab | 36.376 | "Document Title":"health insurance" OR "Abstract":"health insurance" | 364 | Title:("health insurance") OR Abstract:("health insurance") | 96 |
| #49 | ministr*  [Title/Abstract] | 27.106 | TS=("health department*") | 7.787 | (ministr*):ti,ab | 2.201 | "Document Title":"public sectors" OR "Abstract":"public sectors" | 164 | Title:("public sector") OR Abstract:("public sector") | 426 |
| #50 | institute*  [Title/Abstract] | 181.003 | TS=("national health program*") | 541 | (institute*):ti,ab | 20.196 | "Document Title":"public sector" OR "Abstract":"public sector" | 966 | Title:("public sectors") OR Abstract:("public sectors") | 45 |
| #51 | department*  [Title/Abstract] | 334.254 | TS=("health plan*") | 12.753 | (department*):ti,ab | 34.228 | **{OR #32-#50}** | **76.007** | **{OR #32-50}** | **17.520** |
| #52 | “health agency“  [Title/Abstract] | 2.085 | TS=(insurance*) | 133.747 | (“health agency”):ti,ab | 84 | **#19 AND #31 AND #51** | **418** | **#19 AND #31 AND #51** | **73** |
| #53 | “health agencies“  [Title/Abstract] | 4.947 | TS=(reimbursement) | 22.550 | (“health agencies”):ti,ab | 141 |  |  |  |  |
| #54 | “health authority“  [Title/Abstract] | 3.766 | **{OR #34-#52}** | **2.697.509** | (“health authority”):ti,ab | 269 |  |  |  |  |
| #55 | “health authorities“  [Title/Abstract] | 9.730 | **#22 AND #33 AND #54** | **8.227** | (“health authorities”):ti,ab | 370 |  |  |  |  |
| #56 | “public sector“  [Title/Abstract] | 7.328 |  |  | ("public sector"):ti,ab | 371 |  |  |  |  |
| #57 | “public sectors“  [Title/Abstract] | 521 |  |  | ("public sectors"):ti,ab | 9 |  |  |  |  |
| #58 | “health insurance“  [Title/Abstract] | 44.314 |  |  | (“health insurance”):ti,ab | 2.311 |  |  |  |  |
| #59 | “health insurances“  [Title/Abstract] | 455 |  |  | (“health insurances”):ti,ab | 47 |  |  |  |  |
| #60 | “ministry of health“  [Title/Abstract] | 16.651 |  |  | (“ministry of health”):ti,ab | 1553 |  |  |  |  |
| #61 | “health ministry“  [Title/Abstract] | 1.029 |  |  | (“health ministry”):ti,ab | 98 |  |  |  |  |
| #62 | “institute of health“[  Title/Abstract] | 4.778 |  |  | (“institute of health”):ti,ab | 1.040 |  |  |  |  |
| #63 | “health institute“  [Title/Abstract] | 939 |  |  | (“health institute”):ti,ab | 61 |  |  |  |  |
| #64 | “department of health“  [Title/Abstract] | 13.171 |  |  | (“department of health”):ti,ab | 673 |  |  |  |  |
| #65 | “health department“  [Title/Abstract] | 5.963 |  |  | (“health department*”):ti,ab | 298 |  |  |  |  |
| #66 | “Government“[Mesh] | 150.148 |  |  | MeSH descriptor: [Federal Government] explode all trees | 919 |  |  |  |  |
| #67 | “Federal Government“  [Mesh] | 115.676 |  |  | MeSH descriptor: [Government] explode all trees | 983 |  |  |  |  |
| #68 | “Government Agencies“[Mesh] | 128.758 |  |  | MeSH descriptor: [Government Agencies] explode all trees | 955 |  |  |  |  |
| #69 | “State Government“  [Mesh] | 11.937 |  |  | MeSH descriptor: [State Government] explode all trees | 6 |  |  |  |  |
| #70 | “Local Government“  [Mesh] | 3.408 |  |  | MeSH descriptor: [Local Government] explode all trees | 14 |  |  |  |  |
| #71 | “Public Sector“[Mesh] | 6.629 |  |  | MeSH descriptor: [Public Sector] explode all trees | 58 |  |  |  |  |
| #72 | “National Health Programs“[Mesh] | 92.339 |  |  | MeSH descriptor: [National Health Programs] explode all trees | 571 |  |  |  |  |
| #73 | “Regional Health Planning“[Mesh] | 40.074 |  |  | MeSH descriptor: [Regional Health Planning] explode all trees | 275 |  |  |  |  |
| #74 | “Insurance, Health“[Mesh] | 148.570 |  |  | MeSH descriptor: [Insurance, Health] explode all trees | 1.123 |  |  |  |  |
| #75 | “Insurance, Health, Reimbursement“[Mesh] | 46.123 |  |  | MeSH descriptor: [Insurance, Health, Reimbursement] explode all trees | 290 |  |  |  |  |
| #76 | **{OR #46-#75}** | **1.454.483** |  |  | **{OR #46-#71}** | **86.069** |  |  |  |  |
| #77 | **#25 AND #45 AND #76** | **12.086** |  |  | **#25 AND #45 AND #72** | **1.483** |  |  |  |  |
